# First identification of *kdr* allele F1534S in VGSC gene and its association with resistance to pyrethroid insecticides in *Aedes albopictus* populations from Haikou City, Hainan Island, China

**DOI:** 10.1186/s40249-016-0125-x

**Published:** 2016-05-02

**Authors:** Huiying Chen, Kaili Li, Xiaohua Wang, Xinyan Yang, Yi Lin, Fang Cai, Wenbin Zhong, Chunyan Lin, Zhongling Lin, Yajun Ma

**Affiliations:** Department of Tropical Infectious Diseases, Faculty of Tropical Medicine and Public Health, Second Military Medical University, Shanghai, 200433 China; Haikou Center for Disease Control and Prevention, Haikou, 571100 China; CDC Key Laboratory of Surveillance and Early-Warning on Infectious Disease, Haikou, 571100 China

**Keywords:** *Aedes albopictus*, Pyrethroids, Resistance, *kdr* mutation, China

## Abstract

**Background:**

*Aedes albopictus* is distributed widely in China, as a primary vector of Dengue fever and Chikungunya fever in south of China. Chemical insecticide control is one of the integrated programmes to prevent mosquito-borne diseases. Long-term applications of pyrethroids have resulted in the development of resistance in *Ae. albopictus* populations in China. However, the susceptibility of *Ae. albopictus* to pyrethroids in Hainan Island was unclear. Knockdown resistance (kdr), caused by point mutations in the VGSC gene, is one of the mechanisms that confer resistance to DDT and pyrethroids. This study was to investigate the resistance level of *Ae. albopictus* populations in Haikou City to three pyrethroid insecticides, and elucidate the relationship between the resistant phenotype and *kdr* mutations.

**Methods:**

The *Aedes albopictus* samples were collected in Xinbu Island (XI), Longtang Town (LT), Shishan Town (ST), Baishamen Park (BP), and Flower Market (FM) from Haikou City, Hainan Island, China. The larval susceptibility to deltamethrin, permethrin and beta-cypermethrin was tested by larval bioassays, and adult susceptibility to deltamethrin and DDT was determined by adult bioassays. The degree of resistance was determined by resistance ratio value (RR_50_ > 3) for larvae and by mortality for adult. The *kdr* alleles at codon 1534 of the VGSC gene were genotyped. The relationship between *kdr* genotypes and resistant phenotypes was analyzed by Chi-square test.

**Results:**

Out of five populations, assessed by larval bioassays, XI was susceptible to deltamethrin and permethrin; LT was susceptible to permethrin and beta-cypermethrin; and ST was susceptible to permithrin. FM and BP both were resistant to all of the three pyrethroids, and FM showed the highest degree of resistance, with RR_50_ values from 65.17 to 436.36. A total of 493 individuals from the larval bioassays were genotyped for *kdr* alleles. Five alleles were detected, including two wildtype alleles, TTC(F) (67.04 %) and TTT(F) (0.41 %), and three mutant alleles, TGC(C) (0.30 %), TCC(S) (31.54 %) and TTG(L) (0.71 %). There was a clear correlation between mutant alleles (or F1534S) and resistant phenotypes (*P* < 0.01).

**Conclusion:**

Two novel *kdr* mutant alleles F1534S and F1534L were detected in the pyrethroid resistant populations of *Ae. albopictus* in Haikou Hainan, China. For the first time, the mutant F1534S was associated with pyrethroid resistance in *Ae. albopictus.*

**Electronic supplementary material:**

The online version of this article (doi:10.1186/s40249-016-0125-x) contains supplementary material, which is available to authorized users.

## Multilingual abstracts

Please see Additional file 1 for translation of the abstract into the six official working languages of the United Nations.

## Background

*Aedes albopictus* Skuse is a primary vector of Dengue fever and Chikungunya fever in China [1, 2]. Mosquito control is one of the integrated programmes to prevent transmission of mosquito-borne diseases. Chemical insecticides have been extensively used for vector management since the 1940s. There were four major categories of insecticides: organochlorines, organophosphates, carbamates and pyrethroids [3]. The pyrethroids have been used to indoor/outdoor residual sprays since 1980s for mosquito control in China. The long-term utilization has resulted in the development of resistance in many populations of *Ae. albopictus* in China [2, 4–10]. The pyrethroids function as neurotoxins that target voltage-gated sodium channels (VGSC) and interfere electronic signaling in the nervous system, which results in paralysis and death, an effect known as knockdown [11]. One of the mechanisms that mosquitoes have developed for the resistance to pyrethroids is the target insensitivity, which is caused by mutations in the VGSC gene and generated knockdown resistance (*kdr*) [12–15]. In *Anopheles* mosquitoes, substitution of leucine at residue position 1014 was correlated to the resistance to pyrethroids and DDT [14–17]. In *Aedes aegypti* Linn, mutants have been detected in several codons of the VGSC gene from different countries, including three mutants, V1016G/I and F1534C, all were correlated with *kdr* [18–27]. In *Ae. albopictus,* the relationship between *kdr* and pyrethroid resistance was unclear. In a DDT and pyrethroid resistant population of *Ae. albopictus* in India, no *kdr* mutations were detected [28]. Similarly, no *kdr* mutations were found in *Ae. albopictus* populations in Malaysia where F1534C and V1016G/I were detected in the populations of *Ae. aegypti* [29]. So far, only one study has identified the F1534C mutant allele in a population of *Ae. albopictus* in Singapore with frequency of 73.1 % [13].

Haikou city is a provincial capital of Hainan Island, in south of China, located at marginal zone of tropic. In the past, dengue fever outbreaks have occurred twice in 1979–1982 and 1985–1988 in Hainan Island and surrounding areas; the mortality rate was 0.785‰ [30–35]. In recent years, dengue fever epidemic situations remain in Guangdong, Fujian and Yunnan Provinces in China [30, 36–38]. Especially in 2014, a large-scale outbreak of dengue fever with more than 45,000 cases occurred in Guangdong Province [2, 37, 39, 40]. Hainan Island is near to but separated by a strait from Guangdong Province, and there were also reported local cases during the dengue outbreak in 2014 [2]. Upon the pressure of dengue epidemics, residual and aerial spraying of pyrethroids have become a major routine method for the control of *Aedes* mosquitoes in the endemic areas in China. The most commonly used pyrethroid was deltamethrin [2, 41]. Pyrethroid resistance has been detected in the populations of *Ae. aegytpi* and *Ae. albopictus* in Hainan [42, 43]. In this study, we investigated the susceptibility to pyrethroid resistance and examined the *kdr* mutations in *Ae. albopictus* in five locations in Haikou City, Hainan Island. The bioassays revealed that resistance to deltamethrin, permethrin and beta-cypermethrin was developed in certain populations. In addition to the known *kdr* mutant, F1534C, two novel mutant alleles, F1534S and F1534L, were detected.

## Methods

### Ethics statement

No permits were required for the described field studies. Mosquito collections in breeding sites were consent by the owners at each location.

### Mosquito samples

**Fig. 1:**
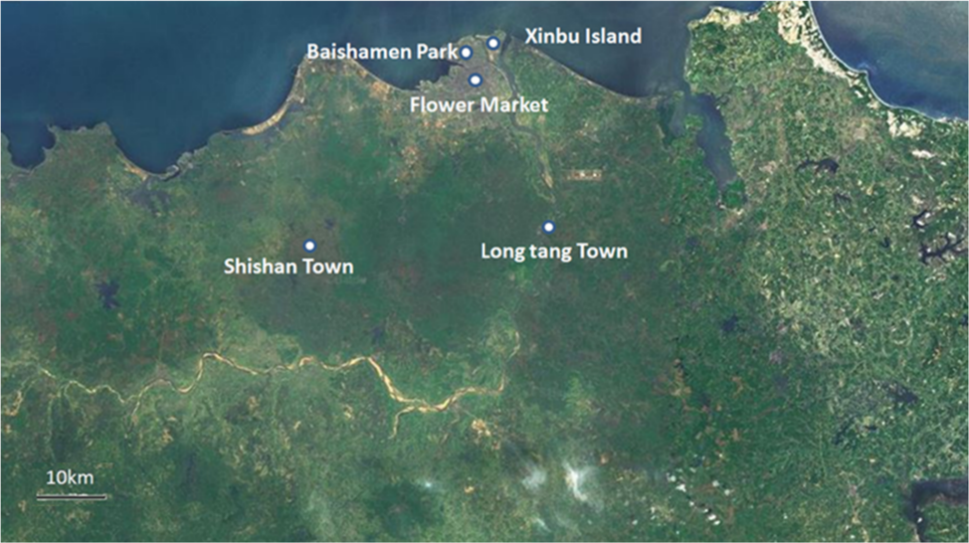
A map of Hainan province (partial) showing the collecting sites

Mosquito larvae were collected from breeding sites in Xinbu Island (XI, 110°37′E, 20°06′N), Longtang Town (LT, 110°42′E, 19°89′N), Shishan Town (ST, 110°22′E, 19°94′N), Baishamen Park (BP, 110°34′E, 20°08′N) and Flower Marker (FM, 110°29′E, 20°02′N) in Haikou city, Hainan Province during April and May 2015 (Fig. 1). The collected larvae were brought back to the insectary and reared to adults at 26 ± 1 °C and 70 ± 5 % (RH), under a 14: 10 h (light: dark) photoperiod. The larvae of F2 generation were used for larval bioassays. The species of *Ae. albopictus* was identified by adult morphology [1]. The susceptible laboratory colony of *Ae. albopictus* was provided by Department of Tropical Infectious Diseases, Second Military Medical University, which was established from a population originally collected from Hangzhou, China. The colony has been maintained in insectary for 15 years without exposure to any insecticides.

### Larval bioassay

The susceptibility of larvae to three pyrethroid insecticides, deltamethrin (≥98 %, Sigma, USA), permethrin (≥98 %, Sigma, USA) and beta-cypermethrin (>99 %, Sigma, USA), was determined using a procedure recommended by WHO [44]. In the assay, 20–25 late 3rd and early 4th instar larvae were placed in a glass container that held 199 mL H_2_O and 1 mL of insecticide solution. Analytical grade insecticides were diluted five to seven concentrations with acetone. The solution with no insecticide was used as control. Larval mortality was recorded 24 h after treatment. The larvae that were motionless or convulsive upon a sharp stimulation were counted as dead [44]. Larval mortality was determined by dividing the number of dead larvae by the total number tested. Dead and survival larvae were collected and preserved in 95 % alcohol for subsequent DNA analysis. No food was provided to larvae during the procedure. If a test with pupation rate greater than 10 %, or mortality rate in control greater than 20 %, the test was invalid and was removed. All bioassays were repeated three times. In the larval bioassay, the median lethal concentration (LC_50_), the 90 % lethal concentration (LC_90_) and 95 % confidence interval of different pyrethroids were calculated based on the recorded data using Schoofs and Willhite’s probit analysis program [45]. The degree of resistance was determined by the resistance ratio (RR_50_), obtained by the LC_50_ value for a population compared with the LC_50_ value for the insecticide for susceptible laboratory colony. The RR_50_ ≤ 3 was considered as susceptible, and 3 < RR_50_ ≤ 10 as low degree of resistance, 10 < RR_50_ ≤ 20 as median degree of resistance, and RR_50_ > 20 as high degree of resistance [44].

### Adult bioassay

Field-collected larvae were reared to adults in the insectary. Female unfed adults at day 2 or 3 post emergence were tested for the susceptibility to deltamethrin and DDT, using the standard WHO tube bioassay [46]. So far, there has been no sufficient data for a standard diagnostic concentration for resistance monitoring for *Ae. albopictus* in China. The test papers with deltamethrin (0.1 %) and DDT (4 %) were used for the assay, which were provided by National Institute for Communicable Disease Control and Prevention, Chinese Center for Disease Control and Prevention. For each insecticide, approximately 100 female mosquitoes were tested. Paraffin oil-treated papers without insecticide were used as control. The knockdown time of individual mosquitoes was recorded at 10 min, 30 min, 50 min and 60 min. Post 1 h exposure, mosquitoes were transferred to a recovery tube and maintained on 6 % of sucrose solution for 24 h. Dead and survival mosquitoes were collected and preserved in 95 % ethanol for subsequent DNA analysis, respectively. Mosquitoes were considered dead if they were motionless, when they were mechanically stimulated, following the method of Gonzalez Audino [47].

### Detection *kdr* alleles and correlation with the larval bioassay

The individual mosquito larvae or adult was used for DNA extraction with the DNAzol Reagent (Invitrogen, USA). To identify *kdr* alleles, a partial sequence of S6 segment of domain III of the VGSC gene was amplified from 20 to 50 ng genomic DNA using primers aegSCF7 (5’-AGG TAT CCG AAC GTT GCT GT-3’) and aegSCR8 (5’-TAG CTT TCA GCG GCT TCT TC-3’) [13]. The PCR kit was from Aidlab, China. PCR reaction was carried out in Verity 96 well 157 Thermal Cycler (Applied Biosystems, USA). The cycling parameter included an initial step of denaturation at 94 °C for 2 min, followed by 35 cycles of amplification at 94 °C for 30 s, 52 °C for 30 s, and 72 °C for 30 s, with a final extension step at 72 °C for 8 min. After electrophoresis, PCR products were purified and directly sequenced in both directions with the same primers. There were 4 specimens, of which the PCR products were cloned into plasmids (pGEMX-T Easy Vector, Aidlab, China), and then sequenced, due to the double peaks at two positions of the codon 1534.

The codon 1534 was examined by sequence analysis, and genotypes were determined. In each sample, for a particular allele, the allele frequency was calculated as: number of alleles/(sample size × 2). The mutation frequency was defined as frequency of sum of wildtype/mutant heterozygotes and mutant/mutant homozygotes, which was calculated as: (sum of wildtype/mutant + mutant/mutant individuals)/sample size.

Chi-square tests were used to examine the association between *kdr* mutation and the resistance phenotype. In the present study, the dependent variables were the mosquito status (alive or dead) at 24 h post larval bioassay.

## Results

### Insecticide susceptibility bioassays

The larval susceptibility to three pyrethroids was tested for five populations of *Ae. albopictus*, which revealed a heterogeneous pattern (Table 1). Among five tested populations, XI (RR_50_ = 2.38), LT (RR_50_ = 1.17) and ST (RR_50_ = 1.67) were susceptible to permethrin; BP was resistant with a median level (RR_50_ = 8.83) and FM was resistant with a high level of resistance (RR_50_ = 182.00). Besides, four of the five populations had developed resistance to deltamethrin and beta-cypermethrin, only XI was susceptible to deltamethrin and LT was susceptible to beta-cypermethrin. FM appeared to be the population having high level of resistance, with RR_50_ = 436.36 to deltamethrin and RR_50_ = 65.17 to beta-cypermethrin (Table 1).

**Table 1:** Susceptibility of *Aedes albopictus* larva to three pyrethroid insecticides in Haikou City, Hainan Island, China

Insecticides	Sites	LC_50_ (mg/L)	LC_50_ (95%CI)	LC_90_ (mg/L)	LC_90_ (95 % CI)	RR_50_
Deltamethrin	XI	0.0001	0.0001–0.0002	0.0003	0.0003–0.0004	1.27
	LT	0.0012	0.0011–0.0014	0.0032	0.0027–0.0040	9.09
	ST	0.0020	0.0010–0.0020	0.0070	0.0050–0.0100	18.18
	BP	0.0080	0.0070–0.0090	0.0210	0.0180–0.0270	72.73
	FM	0.0480	0.0420–0.0550	0.1650	0.1300–0.2320	436.36
	S	0.0001	0.0001–0.0001	0.0003	0.0003–0.0005	
Permethrin	XI	0.0143	0.0134–0.0159	0.0259	0.0232–0.0300	2.38
	LT	0.0070	0.0060–0.0070	0.0120	0.0110–0.0130	1.17
	ST	0.0100	0.0100–0.0110	0.0220	0.0190–0.0270	1.67
	BP	0.0530	0.0490–0.0580	0.1130	0.0990–0.1320	8.83
	FM	1.0920	0.9540–1.2530	4.6740	3.5090–7.1620	182.00
	S	0.0060	0.0050–0.0060	0.0090	0.0080–0.0100	
Beta-cypermethrin	XI	0.0047	0.0043–0.0052	0.0132	0.0114–0.0158	5.31
	LT	0.0020	0.0020–0.0020	0.0040	0.0030–0.0040	2.25
	ST	0.0040	0.0030–0.0040	0.0100	0.0080–0.0120	4.49
	BP	0.0130	0.0120–0.0140	0.0310	0.0260–0.0400	14.61
	FM	0.0580	0.0530–0.0640	0.1740	0.1500–0.2120	65.17
	S	0.0009	0.0008–0.0010	0.0020	0.0020–0.0031	

The data of deltamethrin and permethrin was from the literature [52]

*XI* Xinbu Island, *LT* Longtang Town, *ST* Shishan Town, *BP* Baishamen Park, *FM* Flower Market, *S*: susceptible colony

The adult bioassay was conducted to determine the susceptibility to DDT and deltamethrin. The larvae from the 5 locations were pooled and reared to adults in the insectary. The adults were exposed to the 4 % DDT test paper. The knockdown percentage was 0.00, 0.02, 0.32 and 0.72 % at 10 min, 30 min, 50 min and 60 min. The mortality was 87.50 %, indicating that the population was resistant to DDT. There is no standard diagnostic dosage yet for *Ae. albopictus* adult bioassay in China. The test paper with 0.1 % of deltamethrin was used for testing, which yielded a mortality of 98.40 % in the tested sample (Table 2). The knockdown percentage was 0.32, 0.84, 0.98 and 0.93 % at 4 time nodes,

**Table 2:** *kdr* alleles in relation to mosquito survival phenotype determined by the deltamethrin and DDT susceptibility adult bioassay in *Aedes albopictus* populations in Haikou City, Hainan Island, China

Insecticide	Bioassay			*kdr* alleles					Mutant frequency (%)
	Individuals (N)	Dead (N) after 24 h recovery period	Mortality rate (%)	Bioassay status after 24 h recovery period	Individuals (N)	Wildtype	Mutant		
						TTC(F)	TCC(S)	TGC(C)	
Deltamethrin	104	102	98.40	Alive	2	0	4	0	100.00
				Dead	17	34	0	0	0.00
DDT	198	173	87.50	Alive	19	15	21	2	60.53
				Dead	17	32	2	0	5.89

### Detection of mutant *kdr* gene and correlation with the bioassay

The VGSC gene was genotyped for *kdr* alleles. A total of 493 specimens from larval bioassay samples were typed. At codon 1534, in addition to the wildtype codon TTC encoding phenylalanine (F), four other alleles were detected. Codon TTT codes also for phenylalanine (F), codon TCC codes for serine (S), TGC for cysteiine (C) and TTG for leucine (L). The allele frequency was TTC (F) (67.04 %), TTT (F) (0.41 %) TGC (C) (0.30 %), TCC (S) (31.54 %), and TTG (L) (0.71 %). The most frequent mutant allele was TCC (S) (Table 3). A total of eight genotypes were detected, including wildtype genotype TTC/TTC (57.40 %) and TTC/TTT (0.81 %), wildtype/mutant heterozygotes TTC/TCC (17.85 %), TTC/TTG (0.20 %), TTC/TGC (0.41 %), and mutant genotypes TCC/TCC (21.91 %), TCC/TTG (1.22 %), TCC/TGC (0.20 %). Overall, the frequency of mutant genotypes (S/S, S/L and S/C) was 23.33 %, and the frequency of wildtype/mutant heterozygotes (F/S, F/C and F/L) was 18.46 % (in Additional file 2: Table S1). The mutant frequency was high in both BP and FM while low or none in LT and ST populations of *Ae. albopictus* (Table 3).

**Table 3:** *kdr* alleles in relation to mosquito survival phenotype determined by three pyrethroids larval bioassay groups in Haikou City, Hainan Island, China

Insecticides	Collecting sites	Bioassay status	Individuals (N)	*kdr* alleles					Mutant frequency (%)
				Wildtype		Mutant			
				TTC(F)	TTT(F)	TCC(S)	TGC(C)	TTG(L)	
Deltamethrin	XI	Alive	17	27	0	7	0	0	20.59
		Dead	15	28	0	2	0	0	6.67
	LT	Alive	21	36	2	2	0	2	9.52
		Dead	13	26	0	0	0	0	0.00
	ST	Alive	20	40	0	0	0	0	0.00
		Dead	17	34	0	0	0	0	0.00
	BP	Alive	17	16	0	16	0	2	52.94
		Dead	13	20	0	6	0	0	23.08
	FM	Alive	19	1	0	36	1	0	97.37
		Dead	16	7	0	23	2	0	78.13
Permethrin	XI	Alive	16	28	0	4	0	0	12.50
		Dead	16	29	0	3	0	0	9.38
	LT	Alive	15	28	2	0	0	0	0.00
		Dead	11	22	0	0	0	0	0.00
	ST	Alive	20	40	0	0	0	0	0.00
		Dead	18	36	0	0	0	0	0.00
	BP	Alive	15	9	0	20	0	1	70.00
		Dead	12	14	0	9	0	1	41.67
	FM	Alive	19	2	0	36	0	0	94.74
		Dead	17	9	0	25	0	0	73.53
Beta-cypermethrin	XI	Alive	13	12	0	14	0	0	53.85
		Dead	18	32	0	4	0	0	11.11
	LT	Alive	19	38	0	0	0	0	0.00
		Dead	14	28	0	0	0	0	0.00
	ST	Alive	14	28	0	0	0	0	0.00
		Dead	15	30	0	0	0	0	0.00
	BP	Alive	20	12	0	27	0	1	70.00
		Dead	19	25	0	13	0	0	34.21
	FM	Alive	20	1	0	39	0	0	97.50
		Dead	14	3	0	25	0	0	89.29

*XI* xinbu Island, *LT* Longtang Town, *ST* Shishan Town, *BP* Baishamen Park, *FM* Flower Market

**Fig. 2:**
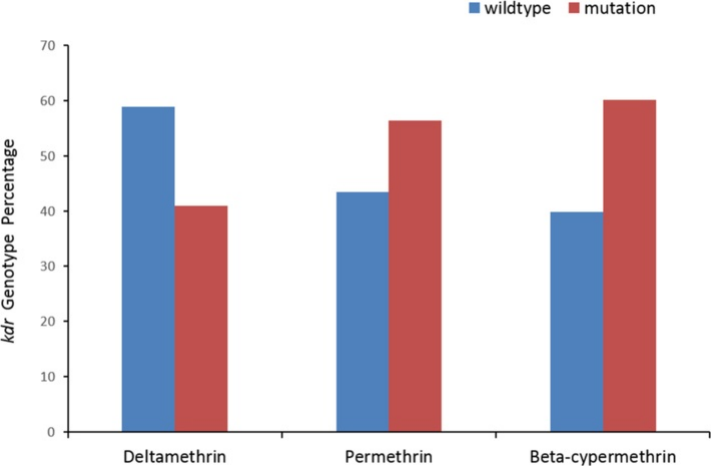
*kdr* genotype percentage in *Aedes albopictus* resistant population to deltamethrin, permethrin and beta-cypermethrin in Haikou City, Hainan Island China

The distributions of wildtype and mutant genotypes in larval populations were shown in Fig. 2. In *Aedes albopictus* resistant population,the frequencies of mutant genotypes were 41.04 % in deltamethrin group, 56.47 % in permethrin group and 60.15 % in beta-cypermethrin group. The frequencies of mutant alleles were 35.11 % in alive individuals and 22.30 % in dead individuals in deltamethrin group, 35.88 % in alive and 25.68 % in dead in permethrin group, 47.09 % in alive and 26.25 % in dead in beta-cypermethrin group. In each case, the mutant alleles were associated with resistant alive mosquitoes (*P* < 0.05). There were all significant differences between the wildtype and mutant alleles in every pyrethroid insecticides bioassay groups (*P* <0.05). The difference was more significant if the individuals from all of the pyrethroid bioassays were pooled together (*P* <0.01).

In the samples from adult bioassay, three alleles were detected, namely TTC (F) (73.64 %), TCC (S) (24.55 %) and TGC (C) (1.82 %), which formed four genotypes: wildtype homozygote TTC/TTC, and wildtype/mutant genotypes, TTC/TGC and TTC/TCC and mutant homozygote TCC/TCC (Table 2). The genotypes of the two resistant mosquitoes that survived the exposure to 0.1 % deltamethrin were both mutant homozygotes of TCC(S). The frequency of mutant alleles was 60.53 % in 19 resistant mosquitoes that survived in the 4 % DDT treatment (Table 2). Significant correlation was detected between *kdr* mutations and deltamethrin or DDT resistant phenotypes by Chi-test (*P* < 0.05).

## Discussion

In Hainan Island, *Aedes* mosquitoes are responsible for the Dengue fever transmissions. The application of ultra low-volume (ULV) spray of pyrethroids has been a major measure to control *Aedes* adults since the 1990s. The susceptibility to pyrethroids has been monitored, and pyrethroid resistance has been reported in wild populations of *Ae. albopictus* in Hainan in 2005 and 2010, respectively [42, 43]. In this study, the larval bioassays showed that the populations in rural areas (XI, ST, LT) were largely susceptible to the pyrethoids tested; while BP and FM, two urban populations, were resistant to all of three pyrethroids. BP represented a population in a city park, where ULV spraying was applied on a regular basis. FM was collected from a garden/nursery market, where containers with aquatic plants, flower pots and planters with sufficient water constitute a large quantity of habitats for *Aedes* larvae. Owners used spray insecticides frequently to reduce mosquito density in the market. In those habitats, mosquitoes expose persistently to high dose of pyrethroids at both larval and adult stages. In rural area, no regular spray was applied, unless dengue patients were present in a village. This may explain why BP and FM mosquitoes were resistant to pyrethroids while the other three rural populations were susceptible.

In the adult assay, adults showed resistance to DDT. When exposed 0.1 % deltamethrin test paper, 98. 40 % of adults were dead. Since the concentration was 4 fold higher than the diagnostic concentration 0.025 % for *Ae. aegypti* [48], we rather not to make any conclusion upon the data. It is an urgent need to develop standard diagnostic concentration for adults of *Ae. albopictus* in China*.*

A number of mutations in the VGSC gene have been reported in pyrethroid resistant strains of *Ae. aegypti* [18–25, 49], a few of these mutations (I1011M/V, V1016G/ I, F1534C) have been clearly associated with the resistance phenotype [12, 20–23, 25]. However, very little is known about the molecular or biochemical basis of resistance in *Ae. albopictus*. No *kdr* mutations were found in *Ae. albopictus* resistant populations from India, Malaysia and Sri Lank [28, 29, 49, 50]. Recently, F1534C was found in 24 of 26 individuals of *Ae. albopictus* in Singapore [13]. In this study, five alleles were identified in the codon 1534, including two wildtype codons, and three mutant codons TCC(S), TGC(C) and TTG(L). The allele TCC(S) was clearly correlated to the resistance to permethrin and beta-cypermethrin, both belong to Type I pyrethroids, similar to the situation in *Ae. aegypti* [51].

This was the first report that *kdr* mutants, particularly F1534S, is behind pyrethroid resistance in *Ae. albopictus.* Apparently, long term applications of DDT and pyrethroids have posed selection pressure on VGSC gene in *Ae. albopictus.* It is required to examine more loci of VGSC gene in more populations in different geographic areas worldwide. In addition, understanding of the resistance mechanisms and development of simple and accurate diagnostic tools to monitor the presence of resistance gene mutations is critical for effective management of pyrethroid resistance and sustainable use of pyrethroid insecticides in the future.

## Conclusions

Some *Ae. albopictus* populations in Haikou City, Hainan Island of China have developed resistance to deltamethrin, permethrin and beta-cypermethrin. The results suggested that *Ae. albopictus* control should adjust the usage of insecticides timely based on the resistant status investigation, and slow down the production and development of resistance. Two novel kdr mutant alleles F1534S and F1534L were detected in the pyrethroid resistant populations of *Ae. albopictus* in Haikou City, Hainan Island of China. For the first time, the mutant F1534S was associated with pyrethroid resistance in *Ae. albopictus*.

## Additional files

Additional file 1: Multilingual abstract in the six official working languages of the United Nations. (PDF 370 kb) Additional file 2: Table S1.
*kdr* genotypes of *Aedes albopictus* populations from pyrethroid larval bioassay groups in Haikou City, Hainan Island, China. **Table S2** Frequencies of *kdr* genotypes in relation to mosquito survival phenotype determined by the deltamethrin and DDT susceptibility adult bioassay in *Aedes albopictus* populations in Haikou City, Hainan Island, China (ZIP 28 kb)

## References

[1] Lu B (1997). Fauna Sinica, Insecta Vol. 9: Diptera, Culicidae I.

[2] Meng F, Wang Y, Feng L, Liu Q (2015). Rivew on dengue prevention and control and integrated mosquito management in China. Chin J Vector Biol Control.

[3] Hemingway J, Ranson H (2000). Insecticide resistance in insect vectors of human disease. Annu Rev Entomol.

[4] Cai R, Shao Z, Fan G, Chen Y (2015). *Aedes albopictus* in different habitats of nine kinds of chemical pesticides resistance research in Huaian City. Chin Pre Med.

[5] Cai S, Duan J, Yin W (2006). Resistance of *Aedes alboptictus* to insecticides and it’s resistance management in Guangdong Province. Chin J Vector Bio Control.

[6] Gong Z, Hou J, Ren Z, Lin F, Guo S (2012). Resistance investigation of *Culex pipiens pallens* and *Aedes albopictus* to eight pesticides and resistance control strategy in Zhejiang province. Chin J Vector Bio Control.

[7] Li C, Hu Z, Jiang Y, Wu H, Luo X, Yan Z (2010). Preliminary Investigation of *Aedes albopictus* resistant to commonly used insecticides in Guangzhou. China Tropical Med.

[8] Li Y, Meng F, Cai S, Liu Q (2013). The resistance of *Aedes albopictus* adult in Zhanjiang city, Guangdong province to deltamethrin and enzyme activity and its characteristics. Chin J Vector Bio Control.

[9] Sun Y, Lv W, Huo L, Zhou Y, Wang B (2013). Insecticide resistance of *Aedes albopictus* in Shaanxi province, China and its control strategy. Chin J Vector Bio Control.

[10] Xu J, Liang X, Yan Z, Hu Z, Jiang Y, Liu C (2014). Resistance of *Aedes albopictus* to three pyrethroids insecticides. Chin J Hyg Insect Equip.

[11] Narahashi T (1996). Neuronal ion channels as the target sites of insecticides. Pharmacol Toxicol.

[12] Kushwah RB, Dykes CL, Kapoor N, Adak T, Singh OP (2015). Pyrethroid-resistance and presence of two knockdown resistance (kdr) mutations, F1534C and a novel mutation T1520I, in Indian *Aedes aegypti*. PLoS Negl Trop Dis.

[13] Kasai S, Ng LC, Lam-Phua SG, Tang CS, Itokawa K, Komagata O (2011). First detection of a putative knockdown resistance gene in major mosquito vector, *Aedes albopictus*. Jpn J Infect Dis.

[14] Wang Y, Yu W, Shi H, Yang Z, Xu J, Ma Y (2015). Historical survey of the *kdr* mutations in the populations of *Anopheles sinensis* in China in 1996–2014. Malar J.

[15] Ibrahim SS, Manu YA, Tukur Z, Irving H, Wondji CS (2014). High frequency of *kdr* L1014F is associated with pyrethroid resistance in *Anopheles coluzzii* in Sudan savannah of northern Nigeria. BMC Infect Dis.

[16] Martinez-Torres D, Chandre F, Williamson MS, Darriet F, Berge JB, Devonshire AL (1998). Molecular characterization of pyrethroid knockdown resistance (kdr) in the major malaria vector *Anopheles gambiae* s.s. Insect Mol Biol.

[17] Aizoun N, Aikpon R, Akogbeto M (2014). Evidence of increasing L1014F kdr mutation frequency in *Anopheles gambiae* s.l. pyrethroid resistant following a nationwide distribution of LLINs by the Beninese National Malaria Control Programme. Asian Pac J Trop Biomed.

[18] Brengues C, Hawkes NJ, Chandre F, McCarroll L, Duchon S, Guillet P (2003). Pyrethroid and DDT cross-resistance in *Aedes aegypti* is correlated with novel mutations in the voltage-gated sodium channel gene. Med Vet Entomol.

[19] Harris AF, Rajatileka S, Ranson H (2010). Pyrethroid resistance in *Aedes aegypti* from Grand Cayman. AmJTrop Med Hyg.

[20] Kawada H, Higa Y, Komagata O, Kasai S, Tomita T, Thi Yen N (2009). Widespread distribution of a newly found point mutation in voltage-gated sodium channel in pyrethroid-resistant *Aedes aegypti* populations in Vietnam. PLoS Negl Trop Dis.

[21] Kawada H, Oo SZ, Thaung S, Kawashima E, Maung YN, Thu HM (2014). Co-occurrence of point mutations in the voltage-gated sodium channel of pyrethroid-resistant *Aedes aegypti* populations in Myanmar. PLoS Negl Trop Dis.

[22] Saavedra-Rodriguez K, Urdaneta-Marquez L, Rajatileka S, Moulton M, Flores AE, Fernandez-Salas I (2007). A mutation in the voltage-gated sodium channel gene associated with pyrethroid resistance in Latin American *Aedes aegypti*. Insect Mol Biol.

[23] Yanola J, Somboon P, Walton C, Nachaiwieng W, Somwang P, Prapanthadara LA (2011). High-throughput assays for detection of the F1534C mutation in the voltage-gated sodium channel gene in permethrin-resistant *Aedes aegypti* and the distribution of this mutation throughout Thailand. Tropical Med int Health TM IH.

[24] Wuliandari JR, Lee SF, White VL, Tantowijoyo W, Hoffmann AA, Endersby-Harshman NM (2015). Association between Three Mutations, F1565C, V1023G and S996P, in the Voltage-Sensitive Sodium Channel Gene and Knockdown Resistance in *Aedes aegypti* from Yogyakarta, Indonesia. Insects.

[25] Chapadense FG, Fernandes EK, Lima JB, Martins AJ, Silva LC, Rocha WT (2015). Phenotypic and genotypic profile of pyrethroid resistance in populations of the mosquito *Aedes aegypti* from Goiania, Central West Brazil. Rev Soc Bras Med Trop.

[26] Vera-Maloof FZ, Saavedra-Rodriguez K, Elizondo-Quiroga AE, Lozano-Fuentes S, Black Iv WC (2015). Coevolution of the Ile1,016 and Cys1,534 Mutations in the Voltage Gated Sodium Channel Gene of *Aedes aegypti* in Mexico. PLoS Negl Trop Dis.

[27] Yanola J, Somboon P, Walton C, Nachaiwieng W, Prapanthadara LA (2010). A novel F1552/C1552 point mutation in the *Aedes aegypti* voltage-gated sodium channel gene associated with permethrin resistance. Pestic Biochem Physiol.

[28] Kushwah RB, Mallick PK, Ravikumar H, Dev V, Kapoor N, Adak TP (2015). Status of DDT and pyrethroid resistance in Indian *Aedes albopictus* and absence of knockdown resistance (kdr) mutation. J Vector Borne Dis.

[29] Ishak IH, Jaal Z, Ranson H, Wondji CS (2015). Contrasting patterns of insecticide resistance and knockdown resistance (kdr) in the dengue vectors *Aedes aegypti* and *Aedes albopictus* from Malaysia. Parasites Vectors.

[30] Du J, Pan X (2010). Prevalent status and features of dengue fever in China. Chin J Epidemiol.

[31] Qiu F, Gubler D, Liu J, Chen Q (1993). Dengue in China: a clinical review. Bull World Health Organ.

[32] Qiu F, Chen Q, Ho Q, Chen W, Zhao Z, Zhao B (1991). The first epidemic of dengue hemorrhagic fever in the People’s Republic of China. AmJTrop Med Hyg.

[33] Fan WF, Yu SR, Cosgriff TM (1989). The reemergence of dengue in China. Rev Infect Dis.

[34] Qiu F, Zhao Z (1988). A pandemic of dengue fever on the Hainan Island. Epidemiol Investig Chin Med J (Engl).

[35] Li F, Yang F, Song J, Gao H, Tang J, Zou C (1986). Etiologic and serologic investigations of the 1980 epidemic of dengue fever on Hainan Island, China. AmJTrop Med Hyg.

[36] Wang W, Yu B, Lin XD, Kong DG, Wang J, Tian JH (2015). Reemergence and Autochthonous Transmission of Dengue Virus, Eastern China, 2014. Emerg Infect Dis.

[37] Ooi EE (2015). The re-emergence of dengue in China. BMC Med.

[38] Lai S, Huang Z, Zhou H, Anders KL, Perkins TA, Yin W (2015). The changing epidemiology of dengue in China, 1990–2014: a descriptive analysis of 25 years of nationwide surveillance data. BMC Med.

[39] Huang L, Luo X, Shao J, Yan H, Qiu Y, Ke P (2016). Epidemiology and characteristics of the dengue outbreak in Guangdong, Southern China, in 2014. Eur J Clin Microbiol Infect Dis.

[40] Li Y, Wu S (2015). Dengue: what it is and why there is more. Sci Bull Sci Found Philipp.

[41] Lu B (1999). Integrated mosquito management Second edition.

[42] Zeng L, Sun D, Zhao W, Li S, Yang X (2010). Determination of the susceptibility of *Aedes aegypti* and *Ae. albopictus* to commonly used insecticides in Hainan province. Chin J Vector Biol Control.

[43] Zeng L, Zhao W, Wang Z, Li S, Yang X (2005). Determination of sensitivity of *Aedes albopictus* and *Aedes aegypi* to pyrethroid indescticides in Hainan Province. China Tropical Med.

[44] WHO (2009). Guidelines for laboratory and field testing of mosquito larvicides.

[45] Schoofs GM, Willhite CC (1984). A probit analysis program for the personal computer. J Appl Toxicol.

[46] WHO (2013). Test procedures for insecticide resistance monitoring in malaria vectors.

[47] Gonzalez Audino P, Vassena C, Barrios S, Zerba E, Picollo MI (2004). Role of enhanced detoxication in a deltamethrin-resistant population of Triatoma infestans (Hemiptera, Reduviidae) from Argentina. Mem Inst Oswaldo Cruz.

[48] Thanispong K, Sathantriphop S, Malaithong N, Bangs MJ, Chareonviriyaphap T (2015). Establishment of Diagnostic Doses of Five Pyrethroids for Monitoring Physiological Resistance in *Aedes Albopictus* in Thailand. J Am Mosq Control Assoc.

[49] Vontas J, Kioulos E, Pavlidi N, Morou E, Torre A, Ranson H (2012). Insecticide resistance in the major dengue vectors *Aedes albopictus* and *Aedes atgypti*. Pestic Biochem Physiol.

[50] Tantely ML, Tortosa P, Alout H, Berticat C, Berthomieu A, Rutee A (2010). Insecticide resistance in *Culex pipiens**quinquefasciatus* and *Aedes albopictus* mosquitoes from La Reunion Island. Insect Biochem Mol Biol.

[51] Hu Z, Du Y, Nomura Y, Dong K (2011). A sodium channel mutation identified in *Aedes aegypti* selectively reduces cockroach sodium channel sensitivity to type I, but not type II pyrethroids. Insect Biochem Mol Biol.

[52] Wang X, Chen H, Yang X, Lin Y, Cai F, Zhong W (2015). Resistance to pyrethroid insecticides and analysis of knockdown resistance (kdr) gene mutations in *Aedes albopictus* from Haikou City. Acad J Sec Mil Med Univ.

