# Supplementary material for: First identification of kdr allele F1534S in VGSC gene and its association with resistance to pyrethroid insecticides in Aedes albopictus populations from Haikou City, Hainan Island, China
Source: Infect Dis Poverty. 2016 May 2;5:31. doi: 10.1186/s40249-016-0125-x (PMC4852438; doi:10.1186/s40249-016-0125-x)
Supplement: Additional file 1: — Multilingual abstract in the six official working languages of the United Nations. (PDF 370 kb) [file 40249_2016_125_MOESM1_ESM.pdf]

Translation of the abstract into the five official working languages of the United Nations

أول تحديد للمقاومة القاضية (kdr) خِلْفة F1534S في جينات VGSC وارتباطه مع المقاومة للمبيدات البيروثرويدات الحشرية في تجمعات بعوضة النمر الأسيوي من مدينة هايكو، جزيرة هاينان، الصين

هوى يينغ تشين، كاي لى لى، شياو هوا وانغ، شين يان يانغ، يي لين، فانغ تساى، ون بن تشونغ، تشون يان لين، تشونغ لينغ لين، يا جون ما

الملخص

الخلفية تنتشر بعوضة النمر الأسيوي على نطاق واسع في الصين، كناقل رئيسي لحمى الضنك وحمى شيكونغونيا في جنوب الصين. والتحكم الكيميائي لإبادة الحشرات هو واحد من البرامج المتكاملة للوقاية من الأمراض التي تنتقل عن طريق البعوض. وقد أدت تطبيقات البيرثرويدات على المدى الطويل في تطوير المقاومة في تجمعات بعوضة النمر الأسيوي في الصين. ومع ذلك، فإن قابلية تأثر بعوضة النمر الأسيوي بالبيرثرويدات في جزيرة هاينان غير واضحة. المقاومة القاضية (KDR)، والناجمة عن طفرات نقطية في الجين VGSC، هي واحدة من الآليات التي تمنح مقاومة للـ دي دي تي والبيريثرويدات. وهذه الدراسة كانت تهدف إلى التعرف على مستوى مقاومة تجمعات بعوضة النمر الأسيوي في مدينة هايكو لثلاثة مبيدات بيروثرويدات حشرية ، وتوضيح العلاقة بين النمط الظاهري وطفرات المقاومة KDR.

الطرق جمعت عينات بعوض النمر الأسيوي في جزيرة تشن بو (XI)، ومدينة لونغ تانغ (LT)، ومدينة شيشان (ST)، وحديقة بايشامن (BP)، وسوق الزهور (FM) من مدينة هايكو، جزيرة هاينان الصينية. وتم اختبار قابلية اليرقات إلى الدلتاميثرين، والبيرميثرين وبيتا- سايبرمثرين بواسطة اختبارات بيولوجية اليرقات، وقابلية الكبار للدلتاميثرين وتم تحديد دي دي تي بواسطة اختبارات بيولوجية الكبار. تم تحديد درجة المقاومة بواسطة قيمة نسبة المقاومة (RR50> 3) لليرقات وبوفيات الكبار. تم التنميط الجيني لخِلْفة المقاومة KDR في كودون 1534 من الجين VGSC. وتم تحليل العلاقة بين مورثات المقاومة القاضية KDR وظواهر المقاومة بواسطة اختبار مربع كاي.

النتائج من بين خمس تجمعات تم تقييمها بواسطة اختبارات بيولوجية اليرقات، كان XI عرضة للدلتاميثرين والبيرميثرين. كان LT عرضة للالبيرميثرين وبيتا- سايبرمثرين. وكان ST عرضة للبيرميثرين. كان FM و BP كلاهما مقاوماً لجميع البيرثرويدات الثلاثة، وأظهرت FM أعلى درجة من المقاومة، مع القيم RR_{50} من 65.17 إلى 436.36. تم التنميط الجيني لإجمالي 493 فرداً من اختبارات بيولوجية اليرقات لخِلْفات المقاومة KDR. تم الكشف عن خمس خِلْفات، من بينهم اثنان من خِلْفات النوع البري، TTC(F) (67.04%) و TTT(F) (0,41%) وثلاثة خِلْفات متحولة، TGC(C) (0.30%)، TCC (S) (31.54%) و TTG (L) (0.71%). كان هناك ارتباط واضح بين الخِلْفات الطافرة (أو F1534S) والأنماط الظاهرية المقاومة (P <0.01).

النتائج تم الكشف عن اثنين جديدين من الخِلْفات المتحولة المقاومة F1534S و F1534L في تجمعات بعوضة النمر الأسيوي المقاومة للبيريثرويد في هايكو هاينان الصينية. وللمرة الأولى، ارتبط F1534S المتحول مع مقاومة البيريثرويد في بعوضة النمر الأسيوي.

Translated from English version into Arabic by Free bird, through

TRANSLATORS
WITHOUT BORDERS

白纹伊蚊海南岛种群中首次发现击倒抗性基因的 F1534S 突变与菊酯类杀虫剂抗药性的相互关系

陈辉莹，李凯利，王晓花，杨新艳，林怡，蔡芳，钟汶兵，林春燕，林忠岭，马雅军

摘要

引言：白纹伊蚊（*Aedes albopictus*）在中国分布广泛，是南方地区登革热和基孔肯雅热的重要传播媒介。使用化学杀虫剂一直是蚊虫防制的主要措施，也是蚊媒病防控的重要环节。长期大量使用拟除虫菊酯类杀虫剂，已导致我国多地白纹伊蚊种群对其产生抗药性，但海南白纹伊蚊的抗药性水平尚不明确。电压门控

钠离子通道（voltage-gated sodium channels, VGSC）编码基因出现点突变会对 DDT 和拟除虫菊酯类杀虫剂产生击倒抗性。本研究的目的是了解海口市白纹伊蚊对 3 种拟除虫菊酯类杀虫剂（溴氰菊酯、氯菊酯和高效氯氰菊酯）抗药性水平，并阐明抗性表型与 *kdr* 基因突变的关系。

方法：本研究在中国海南岛的海口市新埠岛（XI）、龙塘镇（LT）、石山镇（ST）、白沙门（BP）和花卉市场（FM）采集白纹伊蚊样本。应用浸渍法测定幼虫对溴氰菊酯、氯菊酯和高效氯氰菊酯杀虫剂的敏感性，以抗性倍数（RR_{50}）确定抗性级别；应用接触筒法测定成虫对溴氰菊酯和 DDT 的敏感性，以死亡率判定抗性水平。同时对应测定 VGSC 基因的 *kdr* 基因型，卡方检验抗性表型与 *kdr* 突变基因型的关系。

结果：来自 5 个采集地的幼虫中，XI 种群对溴氰菊酯和对氯菊酯均为敏感，LT 种群对氯菊酯和高效氯氰菊酯敏感，ST 种群对氯菊酯敏感，而 FM 和 BP 种群则对 3 种杀虫剂均产生抗性，其中 FM 种群抗性最高，RR_{50} 高达 65.17 至 436.36。对应的 493 只白纹伊蚊幼虫的 *kdr* 基因中，检测到 5 种等位基因，2 种野生型 TTC(F) (67.04%) 和 TTT(F) (0.41%)，3 种突变型 TGC(C) (0.30%)，TCC(S) (31.54%) 和 TTG(L) (0.71%)。突变等位基因与抗性表型之间的关系具有明显统计学意义。

结论：综上所述，在中国海南岛的海口市，白纹伊蚊对拟除虫菊酯类杀虫剂已产生不同水平的抗药性，并检测到白纹伊蚊种群 *kdr* 等位基因新的突变类型 F1534S 和 F1534L，首次记录白纹伊蚊拟除虫菊酯类杀虫剂与抗性相关位点的等位基因 F1534S 存在密切关系。

Translated from English version into Chinese by Chen Hui-Ying

Première identification de l’allèle *kdr* F1534S dans le gène VGSC et association avec une résistance aux insecticides pyréthroïdes auprès des populations de moustiques *Aedes albopictus* de la ville de Haikou, Île de Hainan, Chine

Hui-Ying Chen, Kai-Li Li, Xiao-Hua Wang, Xin-Yan Yang, Yi Lin, Fang Cai, Wen-Bin Zhong, Chun-Yan Lin, Zhong-Ling Lin, Ya-Jun Ma

Résumé

Contexte : le moustique *Aedes albopictus* est largement répandu en Chine et constitue un vecteur primaire de la dengue et du chikungunya dans le sud de la Chine. Le contrôle des insecticides chimiques constitue l’un des programmes intégrés de prévention des maladies transmises par les moustiques. L’utilisation à long terme de pyréthroïdes a entraîné le développement d’une certaine résistance auprès des populations de moustiques *Ae. albopictus* en Chine. La sensibilité du moustique *Ae. albopictus* aux pyréthroïdes était néanmoins incertaine sur l’Île de Hainan. La résistance à l’« effet knockdown » (kdr) due à des mutations ponctuelles du gène VGSC est l’un des mécanismes générant une résistance au DDT et aux pyréthroïdes. La présente étude avait pour objectif d’examiner le niveau de résistance des populations de moustiques *Ae. albopictus* à Haikou aux trois insecticides pyréthroïdes et de déterminer la relation entre le phénotype résistant et des mutations *kdr*.

Méthodes : les spécimens de moustique *Aedes albopictus* ont été recueillis à l’Île de Xinbu (XI), à Longtang (LT), Shishan (ST), au parc Baishamen (BP) et aux marché aux fleurs (FM) de Haikou, Île de Hainan, Chine. La sensibilité larvaire à la deltaméthrine, la perméthrine et la bêta-cyperméthrine a été testée dans le cadre d’essais biologiques au stade larvaire et la sensibilité adulte à la deltaméthrine et au DDT a été déterminée par l’intermédiaire d’essais biologiques menés avec des spécimens adultes. Le degré de résistance a été déterminé par la valeur du rapport de résistance ($RR_{50} > 3$) pour les larves et par la mortalité pour les moustiques adultes. Les allèles *kdr* au niveau du codon 1534 du gène VGSC ont fait l’objet d’un génotypage. La relation entre les génotypes *kdr* et les phénotypes résistants a été analysée dans le cadre d’un test de chi carré.

Résultats : sur les cinq populations évaluées dans le cadre d'essais biologiques au stade larvaire, la population XI était sensible à la deltaméthrine et à la perméthrine, alors que la population LT était sensible à la perméthrine et à la bêta-cyperméthrine et la population ST était sensible à la perméthrine. Les populations FM et BP étaient toutes deux résistantes à l'ensemble des trois pyréthroïdes et la population FM affichait le plus haut niveau de résistance avec des valeurs RR_{50} allant de 65,17 à 436,36. Au total, 493 spécimens issus des essais biologiques réalisés au stade larvaire ont fait l'objet d'un génotypage pour identifier la présence d'allèles *kdr*. Cinq allèles ont été détectés, y compris deux allèles de type sauvage, TTC(F) (67,04 %) et TTT(F) (0,41 %), ainsi que trois allèles mutants, TGC(C) (0,30 %), TCC(S) (31,54 %) et TTG(L) (0,71 %). Il existait une corrélation claire entre des allèles mutants (ou F1534S) et des phénotypes résistants ($P<0,01$).
Conclusion : deux nouveaux allèles *kdr* mutants F1534S et F1534L ont été détectés dans les populations de moustiques *Ae. albopictus* résistants aux pyréthroïdes à Haikou (Chine). Le mutant F1534S a pour la première fois été associé à une résistance aux pyréthroïdes chez les moustiques *Ae. albopictus*.

Translated from English version into French by eric ragu, through

Первое выявление резистентности к нокдаун-эффекту гена в гене VGSC и его связь с резистентностью к перитроидными инсектицидами у популяции Aedes albopictus из города Хаукоу, остров Хайнань, Китай.

Хю-Жин Чен, Кай-Ли ли, Джяо-Хуа Жан, Жи Лин, Фан Кай, Вен-Бин Жонг, Хун-Янь Лин, Жонг-Линг Лин, Йа-Жунь Ма

Краткое описание ситуации

Общие сведения *Aedes albopictus* широко распространён в Китае, являясь основным переносчиком лихорадки Денвера и лихорадки чикунгунья на юге Китая. Контроль химических инсектицидов является одной из интегрированных программ предотвращения заоболеваний, передаваемых комарами. Долгосрочное применение перитроидов вызвало развитие резистенции у популяции *Ae. albopictus* to в Китае. Однако, восприимчивость *Ae. albopictus* to к пиретроидам на острове Хайнань не была установлена. Резистентность к нокдаун-эффекту, вызванная точечной мутацией в гене VGSC, является одним из механизмов, который обеспечивает устойчивать к ДДТ и пиретроидам. Целью этого исследования было изучить устойчивость популяции *Ae. albopictus* to в городе Хаукоу к трём пиретроидным инсектицидам, и прояснить связь между резистенстным фенотипом и мутациями, обеспечивающими резистентность к нокдаун эффекту.
Методы. Образцы *Ae. albopictus* были собраны на остове Чинбу (Xinbu Island (XI)), городе Лонгтанг (Longtang Town (LT)), в городе Шишан (Shishan Town (ST)), парке Байшамень (Baishamen Park (BP)) и на Цветочном Рынке (Flower Market (FM)) к городе Хаукоу, остров Хайнань, Китай. Устойчивать личинок к дельтаметрину, перметрину и бета-циперметрину была исследована в биологических опытах над личинками, а устойчивость взрослых особей к дельтаметриу и ДДТ была определена в биологических опытах над взрослыми опытами. Степень устойчивости определялась показателем доли устойчивости

(RR_{50} > 3) для личинок и смертности для взрослых особей. Был выявлен генотип аллеломорфа, резистентного к нокдаун-эффекту для кодона 1534 гена VGSC. Соотношение между генотипом, резистентным к нокдаун-эффекту, и резистентными фенотипами было проанализировано по критерию хи-квадрат.

Результаты. Из пяти популяций, исследованных в биологических опытах над личинками, XI была восприимчива к дельтаметрину и перметрину; LT была восприимчива к перметрину и бета-циперметрину; а ST была восприимчива к перметрину. FM и BP были обе резистентны ко всем трём пиретроидам, а FM продемонстрировала самую высокую степень устойчивости, с показателями RR_{50} от 65.17 до 436.16. Все 493 особи из биологических опытов с личинками были типизированы как аллеломорфы, резистентные к нокдаун-эффекту. Было выявлено пять аллеломорфов, включая два немутантных аллеломорфа, TTC(F) (67.04%) и TTT(F) (0.41%), и три мутантных аллемофорфа TGC(C) (0.30%), TCC(S) (31.54%) и TTG(L) (0.71%). Была установлена чёткая связь между мутантными аллеломорфами (или F1534S) и резистентными фенотипами ($P < 0.01$).

Вывод. Новые мутантные аллеломорфы F1534S и F1534L были выявлены среди популяции *Ae. albopictus, резистентной к нокдаун-эффекту, в г.Хаукоу, Хайнань, Китай.* Впервые мутант F1534S был соотнесён с пиретроидной резистентностью у *Ae. albopictus.*

Translated from English version into Russian by Tatiana Glazina, through

TRANSLATORS
WITHOUT BORDERS

Alelo Kdr F1534S detectado por primera vez en el gen VGSC y su relación con la resistencia a los piretroides en poblaciones de *Aedes albopictus* de Haikou, provincia de Hainan, China

Hui-Ying Chen, Kai-Li Li, Xiao-Hua Wang, Xin-Yan Yang, Yi Lin, Fang Cai, Wen-Bin Zhong, Chun-Yan Lin, Zhong-Ling Lin, Ya-Jun Ma

Resumen

Antecedentes El *Aedes albopictus* está ampliamente extendido en China como vector principal del dengue y la chicunguña en el sur del país. El control con insecticidas químicos es uno de los programas integrados para prevenir las enfermedades transmitidas por mosquitos. El uso continuado de piretroides ha provocado el desarrollo de resistencia en las poblaciones de *Aedes albopictus* de China. No obstante, la sensibilidad del *Aedes Albopictus* a los piretroides en la provincia de Hainan no estaba del todo clara. La resistencia 'knockdown'(kdr) a los piretroides causada por mutaciones puntuales en el gen VGSC es uno de los mecanismos que confieren resistencia al DDT. El objeto del presente estudio ha sido investigar el grado de resistencia de las poblaciones de *Aedes albopictus* en la ciudad de Haikou a tres piretroides, así como esclarecer la relación existente entre el fenotipo resistente y las mutaciones *Kdr* .

Métodos Las muestras de *Aedes albopictus* se han recogido la isla Xinbu (XI), Longtang Town (LT), Shishan Town (ST), Baishamen Park (BP), y el Mercado de las flores (FM) de Haikou, en la provincia de Hainan, China. La sensibilidad de las larvas a la deltametrina, permetrina y beta-cipermetrina ha sido probada mediante bioanálisis larvales. La sensibilidad de los adultos a la deltametrina y al DDT se ha establecido mediante bioanálisis a adultos.

El grado de resistencia se ha establecido con un valor de la ratio de resistencia de ($RR_{50} > 3$) para las larvas y por la mortalidad para los adultos. Se han genotipado los alelos *kdr* en el codón 1534 del gen VGSC. La relación existente entre los genotipos *Kdr* y los fenotipos resistentes se ha analizado mediante la prueba de la $\chi 2$.

Resultados De cinco poblaciones evaluadas con bioanálisis larvales, la de XI resultó ser sensible a la deltametrina y a lapermetrina; la de LT era sensible a la permetrina y a la beta-cipermetrina y la de ST a la permetrina. Tanto la de FM como la de BP eran resientes a los tres piretroides y FM mostró el grado más elevado de resistencia con valores de RR_{50} de entre 65,17 y 436,36. Un total de 493 individuos procedentes de los bioanálisis larvales han sido genotipados en busca de alelos *Kdr*. Se han detectado cinco alelos, dos silvestres: TTC(F) (67.04%) y TTT(F) (0.41%) y tres mutantes TGC(C) (0.30%), TCC(S) (31.54%) y TTG(L) (0.71%). Había una correlación evidente entre los alelos mutantes (o F1534S) y los fenotipos resistentes ($P < 0.01$).

Conclusión Se han detectado dos nuevos alelos mutantes *Kdr* F1534S y F1534L en poblaciones de *Aedes. albopictus* resistentes a los piretroides en Haikou, provincia de Hainan, China. Por primera vez, la mutación F1534S ha sido relacionada con la resistencia a los piretroides del *Aedes albopictus.*

Translated from English version into Spanish by Raquel Bentué, through

TRANSLATORS WITHOUT BORDERS
