# Supplementary material for: First identification of kdr allele F1534S in VGSC gene and its association with resistance to pyrethroid insecticides in Aedes albopictus populations from Haikou City, Hainan Island, China
Source: Infect Dis Poverty. 2016 May 2;5:31. doi: 10.1186/s40249-016-0125-x (PMC4852438; doi:10.1186/s40249-016-0125-x)
Supplement: Additional file 2: Table S1. — kdr genotypes of Aedes albopictus populations from pyrethroid larval bioassay groups in Haikou City, Hainan Island, China. Table S2 Frequencies of kdr genotypes in relation to mosquito survival phenotype determined by the deltamethrin and DDT susceptibility adult bioassay in Aedes albopictus populations in Haikou City, Hainan Island, China (ZIP 28 kb) [file 40249_2016_125_MOESM2_ESM.zip › Updated Additional file 2/Table S1 kdr genotypes of Aedes albopictus populations from pyrethroid larval bioassay groups in Haikou City, Hainan Island, China.docx]

**Table S1 *kdr* genotypes of *Aedes albopictus* populations from pyrethroid larval bioassay groups in Haikou City, Hainan Island, China**

| Insecticides | Sampling  sites | Phenotype | Individuals (N) | Wildtype genotype | | Wildtype/mutant heterozygote | | | Mutant genotype | | | Mutant Frequency  (%) |
| --- | --- | --- | --- | --- | --- | --- | --- | --- | --- | --- | --- | --- |
|  |  |  |  | TTC(F)/  TTC(F) | TTC(F)/  TTT(F) | TTC (F)/  TCC(S) | TTC(F)/  TGC(C) | TTC(F)/  TTG(L) | TCC(S)/  TCC(S) | TCC(S)/  TTG(L) | TCC(S)/  TGC(C) |  |
| Deltamethrin | XI | Alive | 17 | 10 | 0 | 7 | 0 | 0 | 0 | 0 | 0 | 41.18 |
|  |  | Dead | 15 | 13 | 0 | 2 | 0 | 0 | 0 | 0 | 0 | 13.33 |
|  | LT | Alive | 21 | 17 | 2 | 0 | 0 | 0 | 0 | 2 | 0 | 9.52 |
|  |  | Dead | 13 | 13 | 0 | 0 | 0 | 0 | 0 | 0 | 0 | 0.00 |
|  | ST | Alive | 20 | 20 | 0 | 0 | 0 | 0 | 0 | 0 | 0 | 0.00 |
|  |  | Dead | 17 | 17 | 0 | 0 | 0 | 0 | 0 | 0 | 0 | 0.00 |
|  | BP | Alive | 17 | 4 | 0 | 8 | 0 | 0 | 3 | 2 | 0 | 76.47 |
|  |  | Dead | 13 | 7 | 0 | 6 | 0 | 0 | 0 | 0 | 0 | 46.15 |
|  | FM | Alive | 19 | 0 | 0 | 1 | 0 | 0 | 17 | 0 | 1 | 100.00 |
|  |  | Dead | 16 | 1 | 0 | 3 | 2 | 0 | 10 | 0 | 0 | 93.75 |
| Permethrin | XI | Alive | 16 | 12 | 0 | 4 | 0 | 0 | 0 | 0 | 0 | 25.00 |
|  |  | Dead | 16 | 14 | 0 | 1 | 0 | 0 | 1 | 0 | 0 | 12.5. |
|  | LT | Alive | 15 | 13 | 2 | 0 | 0 | 0 | 0 | 0 | 0 | 0.00 |
|  |  | Dead | 11 | 11 | 0 | 0 | 0 | 0 | 0 | 0 | 0 | 0.00 |
|  | ST | Alive | 20 | 20 | 0 | 0 | 0 | 0 | 0 | 0 | 0 | 0.00 |
|  |  | Dead | 18 | 18 | 0 | 0 | 0 | 0 | 0 | 0 | 0 | 0.00 |
|  | BP | Alive | 15 | 1 | 0 | 7 | 0 | 0 | 6 | 1 | 0 | 93.33 |
|  |  | Dead | 12 | 5 | 0 | 3 | 0 | 1 | 3 | 0 | 0 | 58.33 |
|  | FM | Alive | 19 | 0 | 0 | 2 | 0 | 0 | 17 | 0 | 0 | 100.00 |
|  |  | Dead | 17 | 1 | 0 | 7 | 0 | 0 | 9 | 0 | 0 | 94.12 |
| Beta-cypermethrin | XI | Alive | 13 | 2 | 0 | 8 | 0 | 0 | 3 | 0 | 0 | 84.62 |
|  |  | Dead | 18 | 14 | 0 | 4 | 0 | 0 | 0 | 0 | 0 | 22.22 |
|  | LT | Alive | 19 | 19 | 0 | 0 | 0 | 0 | 0 | 0 | 0 | 0.00 |
|  |  | Dead | 14 | 14 | 0 | 0 | 0 | 0 | 0 | 0 | 0 | 0.00 |
|  | ST | Alive | 14 | 14 | 0 | 0 | 0 | 0 | 0 | 0 | 0 | 0.00 |
|  |  | Dead | 15 | 15 | 0 | 0 | 0 | 0 | 0 | 0 | 0 | 0.00 |
|  | BP | Alive | 20 | 1 | 0 | 10 | 0 | 0 | 8 | 1 | 0 | 95.00 |
|  |  | Dead | 19 | 7 | 0 | 11 | 0 | 0 | 1 | 0 | 0 | 63.16 |
|  | FM | Alive | 20 | 0 | 0 | 1 | 0 | 0 | 19 | 0 | 0 | 100.00 |
|  |  | Dead | 14 | 0 | 0 | 3 | 0 | 0 | 11 | 0 | 0 | 100.00 |
| Total | | | 493 | 283 | 4 | 88 | 2 | 1 | 108 | 6 | 1 | 41.78 |
