# Supplementary material for: First identification of kdr allele F1534S in VGSC gene and its association with resistance to pyrethroid insecticides in Aedes albopictus populations from Haikou City, Hainan Island, China
Source: Infect Dis Poverty. 2016 May 2;5:31. doi: 10.1186/s40249-016-0125-x (PMC4852438; doi:10.1186/s40249-016-0125-x)
Supplement: Additional file 2: Table S1. — kdr genotypes of Aedes albopictus populations from pyrethroid larval bioassay groups in Haikou City, Hainan Island, China. Table S2 Frequencies of kdr genotypes in relation to mosquito survival phenotype determined by the deltamethrin and DDT susceptibility adult bioassay in Aedes albopictus populations in Haikou City, Hainan Island, China (ZIP 28 kb) [file 40249_2016_125_MOESM2_ESM.zip › Updated Additional file 2/Table S2 Frequencies of kdr genotypes in relation to mosquito survival phenotype.docx]

**Table S2 Frequencies of *kdr* genotypes in relation to mosquito survival phenotype determined by the deltamethrin and DDT susceptibility adult bioassay in *Aedes albopictus* populations in Haikou City, Hainan Island, China**

| Insecticide | Bioassay | | *kdr* genotype | | | | | | |
| --- | --- | --- | --- | --- | --- | --- | --- | --- | --- |
|  | Individuals (N) | Mortality rate | Bioassay status | Individuals (N) | Wildtype | Mutant heterozygote / homotozygote | | | Mutant Frequency (%) |
|  |  |  |  |  | TTC(F)/  TTC(F) | TTC(F)/  TGC(C) | TTC(F)/  TCC(S) | TCC(S)/  TCC(S) |  |
| Deltamethrin | 104 | 98.40% | Resistant | 2 | 0 | 0 | 0 | 2 | 100.00 |
|  |  |  | Susceptible | 17 | 17 | 0 | 0 | 0 | 0.00 |
| DDT | 198 | 87.50% | Resistant | 19 | 1 | 2 | 11 | 5 | 94.74 |
|  |  |  | Susceptible | 17 | 16 | 0 | 0 | 1 | 5.88 |

“Resistant” refers to the mosquitoes that were alive 24 hours after 60-min exposure to the insecticides in the standard WHO tube bioassay; and “susceptible” refers to the mosquitoes that were knocked down within the 24 hours recovery period.
